# Heart Rate Variability Mainly Relates to Cognitive Executive Functions and Improves Through Exergame Training in Older Adults: A Secondary Analysis of a 6-Month Randomized Controlled Trial

**DOI:** 10.3389/fnagi.2020.00197

**Published:** 2020-07-15

**Authors:** Patrick Eggenberger, Simon Annaheim, Kerstin A. Kündig, René M. Rossi, Thomas Münzer, Eling D. de Bruin

**Affiliations:** ^1^Empa, Swiss Federal Laboratories for Materials Science and Technology, Laboratory for Biomimetic Membranes and Textiles, St. Gallen, Switzerland; ^2^Department of Health Sciences and Technology, Institute of Human Movement Sciences and Sport, ETH Zurich, Zurich, Switzerland; ^3^Geriatrische Klinik St. Gallen, St. Gallen, Switzerland; ^4^Department of Geriatric Medicine, University of Zurich, Zurich, Switzerland; ^5^Department of Neurobiology, Care Sciences and Society, Karolinska Institutet, Stockholm, Sweden

**Keywords:** cognitive–motor training, dual-task training, normalized heart rate variability, functional fitness, executive functions, verbal long-term memory, gait variability, elderly

## Abstract

Heart rate variability (HRV) mirrors autonomic nervous system activities and might serve as a parameter to monitor health status in older adults. However, it is currently unknown which functional health measures, including cognitive, physical, and gait performance parameters, are most strongly related to HRV indices. This knowledge would enable implementing HRV assessments into health monitoring routines and training planning for older adults. Simultaneous cognitive–motor and exergame training may be effective to improve HRV indices but has not been investigated yet. Eighty-nine healthy older adults (≥70 years of age) were randomized into three groups: (1) virtual reality video game dancing, i.e., exergaming (DANCE); (2) treadmill walking with simultaneous verbal memory training (MEMORY); or (3) treadmill walking only (PHYS). Strength and balance exercises complemented each program. Over 6 months, two weekly 1-h training sessions were performed. HRV indices (standard deviation of N–N intervals, SDNN; root mean square of successive R–R interval differences, RMSSD; and absolute power of high-frequency band (0.15–0.4 Hz), HF power) and various measures of cognitive, physical, and gait performance were assessed at baseline and after 3 months and 6 months. Multiple linear regression analyses with planned comparisons were calculated. At baseline, 8–12% of HRV variance was significantly explained by cognitive executive functions and leg strength (inversely related). Verbal long-term memory, aerobic and functional fitness, and gait performance did not contribute to the model (SDNN: *R^2^* = 0.082, *p* = 0.016; RMSSD: *R^2^* = 0.121, *p* = 0.013; HF power: *R^2^* = 0.119, *p* = 0.015). After 6 months, DANCE improved HRV indices, while MEMORY and PHYS did not (time × intervention interactions: first-contrast DANCE/MEMORY vs. PHYS: SDNN *p* = 0.014 one-tailed, Δ*R*^2^ = 0.020 and RMSSD *p* = 0.052 one-tailed (trend), Δ*R*^2^ = 0.007; second-contrast DANCE vs. MEMORY: SDNN *p* = 0.002 one-tailed, Δ*R*^2^ = 0.035, RMSSD *p* = 0.017 one-tailed, Δ*R*^2^ = 0.012, and HF power *p* = 0.011 one-tailed, Δ*R*^2^ = 0.013). We conclude that mainly cognitive executive functions are associated with HRV indices and that exergame training improves global and parasympathetic autonomic nervous system activities in older adults. Periodic assessments of HRV in older citizens could be particularly beneficial to monitor cognitive health and provide indications for preventative exercise measures.

## Introduction

Health monitoring that aims to introduce preventative strategies is increasingly important in the rapidly growing population of older citizens. Adequate and accurate health monitoring approaches could thereby help to reduce individual suffering and overall healthcare costs. A promising and easily measurable parameter to monitor current health status and predict future health outcomes of older adults is heart rate variability (HRV). HRV indices mirror global, sympathetic, and parasympathetic activities of the autonomic nervous system (Malik, 1996). Low HRV values are usually indicative of compromised health and increased mortality (Ernst, 2017; Kemp et al., 2017). It has been proposed that physiological systems that oscillate within a range of states (which is the case for high HRV) can adapt flexibly to various inputs, whereas dysregulated systems are locked in a specific pattern (similar to low HRV; Thayer et al., 2012). As such, HRV measures may reflect the adaptivity of the brain–body system (Ernst, 2017). Furthermore, HRV indices are associated with numerous domains of cognitive and physical functioning in older adults (Albinet et al., 2010; Ogliari et al., 2015; Freitas et al., 2018).

Different domains of *cognitive functioning*, including global cognition, executive functions, reaction time, and processing speed, are related to HRV indices in older adults. Frewen et al. (2013) reported that older adults with lower HRV recordings also performed worse in a global cognition test, independently of confounders. Similarly, reduced HRV values in older adults were associated with lower performance in global cognitive functioning (Al Hazzouri et al., 2014). A recent meta-analysis indicated that HRV indices may be applied as biomarkers of top-down self-regulation processes, such as executive functioning, emotion regulation, and effortful control (Holzman and Bridgett, 2017). Additionally, lower resting 10-second HRV recordings were linked to slower reaction times and processing speed in older adults independently of possible confounders including medication, cardiovascular risk factors, and comorbidities (Mahinrad et al., 2016).

Reduced HRV, particularly the index standard deviation of normal-to-normal beat intervals (SDNN), is related to poorer *physical functional status* in activities of daily living (ADL) and predicts the risk of future functional decline of older adults, as reported in a study with 5,042 participants (Ogliari et al., 2015). Low physical capabilities, assessed with functional tests, including single-leg stance for balance, gait speed, and chair rises for leg strength, were associated with decreased HRV values in obese older adults (Liao et al., 2017). Additionally, HRV data (SDNN) predicted longevity in persons at very old age (86 ± 14 years), in contrast to an annual health examination that was not associated with this outcome (Kurita et al., 2013). HRV is also a good predictor of mortality due to chronic heart failure, which is a condition related to autonomic dysfunction (Nolan et al., 1998). Furthermore, HRV appears to be sensitive to *exercise training-induced adaptations* and, therefore, may be suited to monitor beneficial effects of preventative and therapeutic interventions. Particularly, aerobic exercise training was repeatedly shown to modulate HRV indices in older adults. A recent meta-analysis containing 12 studies and 329 participants beyond age 60 years demonstrated positive effects of aerobic exercise training on the global HRV index SDNN. Thereby, improvements in HRV were linearly increasing with higher exercise frequency (Raffin et al., 2019).

Supported by this previous research, HRV indices seem to provide a promising measure to assess cognitive and physical functional health risk factors in early preclinical phases. Hence, HRV monitoring may enable the early adoption of preventative interventions, such as exercise and cognitive training. In addition, it provides a measure that is sensitive to follow-up exercise training-induced adaptations.

However, thus far it remains unknown how much of the variance in HRV indices is associated with different cognitive, physical, and gait performance parameters, reflecting functional health of older adults. This knowledge would be important in order to implement HRV measures into a monitoring routine for older adults at risk for developing cognitive or physical health issues and inform about the potentially important exercise programming components. Exercise-training modalities that simultaneously incorporate both cognitive and motor aspects can be hypothesized to have a different effect compared to exercise programs targeting only physical components. However, such combined cognitive–motor programs have not yet been investigated in relation to HRV in older adults. Based on the interconnection of HRV with cognitive and physical health factors outlined above, combined cognitive–motor training through exergame training potentially bears larger effects on HRV indices compared to exclusively physical (aerobic) training.

Therefore, the *aims* of this study were: (1) to investigate, in a *cross-sectional analysis*, which cognitive, physical, and gait parameters explain the variance in HRV indices at baseline; and (2) to investigate in a *longitudinal analysis* whether cognitive–motor training induces different adaptations in HRV indices compared to exclusively physical training.

We *hypothesized*: (1) that variance in HRV indices is explained by; (a) cognitive parameters related to executive functions and verbal memory; (b) physical parameters related to physical functioning, aerobic endurance, and leg strength; and (c) gait parameters related to executive functions and, furthermore, (2) that exercise training-induced adaptations in HRV indices primarily occur in combined cognitive–motor training interventions and particularly in training forms which include aspects of executive functioning (i.e., exergame training).

## Materials and Methods

### Study Design and Participants

**Figure 1:**
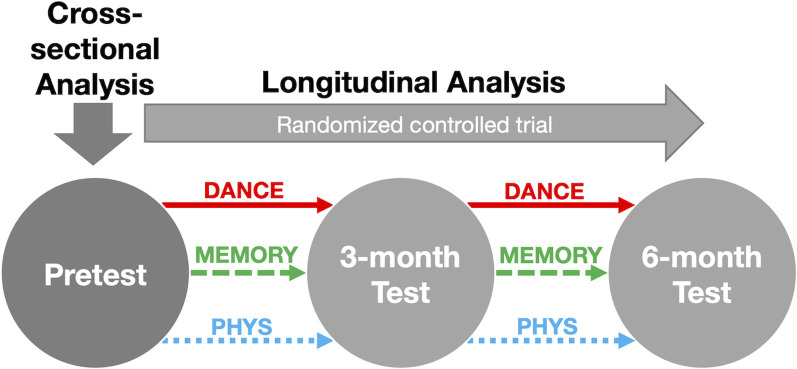
Overview of the study design. Notes: assessments of HRV indices, cognitive, physical, and gait performance were conducted at each test time-point. Abbreviations: DANCE, virtual reality video game dancing; HRV, heart rate variability; MEMORY, treadmill walking with simultaneous verbal memory training; PHYS, treadmill walking.

This is a secondary data analysis of a randomized controlled trial, comprising a 6-month training intervention and three parallel intervention groups (Eggenberger et al., 2015a,b). Measurements were performed at baseline (pre-training) and after 3 (mid-training) and 6 months (post-training), respectively (Figure 1). Data collection and training took place at Geriatrische Klinik, St. Gallen, Switzerland. The ethics committee of the Canton St. Gallen, Switzerland, approved the study protocol (EKSG 12/092), and the study was registered at Current Controlled Trials ISRCTN70130279. The planned methods remained unchanged after trial commencement. Reporting of this study adheres to the CONSORT guidelines (Moher et al., 2010).

Healthy older adults (≥70 years of age) were recruited from the community and residential homes between August until September 2012. The training intervention started in October 2012 and ended at the end of March 2013. Details on recruitment procedures, eligibility criteria, *a priori* sample size estimation, and randomization are summarized previously (Eggenberger et al., 2015a,b). Related to the eligibility criteria, particularly older adults with health conditions such as dementia, stroke, or recent head injury that could potentially confound the results of cognitive performance outcomes were excluded.

### Training

Training sessions were performed twice weekly for 1 h with a pause of 1–2 days to allow recovery. Fifty-two training sessions were completed within 6 months (26 weeks). Each of the three intervention groups’ sessions contained 20 min of strength and 20 min of balance training based on current recommendations for physical fitness and fall prevention for older adults (American College of Sports Medicine et al., 2009; Sherrington et al., 2011; Granacher et al., 2012). Additionally, the DANCE group performed 20 min of virtual reality video game dancing, i.e., exergaming (Impact Dance Platforms, Positive Gaming BV, Haarlem, the Netherlands; StepMania Software), the MEMORY group performed 20 min of treadmill walking with concurrent memory training (presented on a computer screen), and the PHYS group performed 20 min of treadmill walking only. This resulted in two training intervention arms including simultaneous cognitive–motor training (DANCE and MEMORY) and one training intervention arm comprising exclusively physical training (PHYS), which served as an active control group. Thereby, the virtual reality video game dance training combines cognitive aspects of attention and executive functions with motor coordination, whereas the treadmill walking memory training comprised verbal memory exercises. Further details of each training intervention were reported elsewhere (Eggenberger et al., 2015a,b).

### Measurements

#### Heart Rate Variability (Primary Outcome)

HRV was assessed before the 6-min-walk test (6-MWT). Participants were instructed to sit in a comfortable position on a chair without speaking for 5 min, which is the standard time period for short-term recordings (Malik, 1996). During this time, beat-to-beat R–R intervals were recorded by means of a wearable heart rate monitor and chest belt (RS800CX Polar Electro Oy, Kempele, Finland). This device provides valid and reliable test–retest HRV recordings (Williams et al., 2017). Based on the R–R intervals, the following eight HRV indices were calculated: the time-domain indices SDNN (ms), pNN50 (percentage of adjacent normal-to-normal intervals that differ by more than 50 ms, %), and RMSSD (root mean square of successive R–R interval differences, ms); the frequency-domain indices LF power (absolute power of low-frequency band, ms^2^, 0.04–0.15 Hz), HF power (absolute power of high-frequency band, ms^2^, 0.15–0.4 Hz), and the ratio of LF to HF power (%); and non-linear indices SD1 (Poincaré-plot standard deviation perpendicular to the line of identity, ms) and SD2 (Poincaré-plot standard deviation along the line of identity, ms). SDNN is an estimate of overall or global HRV and is influenced by both the sympathetic and parasympathetic branches of the autonomic nervous system; LF power and SD2 are not only pure measures of sympathetic drive but also affected by parasympathetic activity; and the LF/HF ratio intends to estimate the balance between sympathetic and parasympathetic effects, whereas pNN50, RMSSD, HF power, and SD1 represent high-frequency variation in heart rate and are interpreted as proxy for the activity in the parasympathetic or vagally mediated division of the autonomic nervous system (Malik, 1996; Ernst, 2017; Shaffer and Ginsberg, 2017).

#### Secondary Outcome Measures

Seven *cognitive performance tasks*, which cover different cognitive domains, were chosen to identify associations with HRV indices. These “paper-and-pencil” tasks included the Trail Making Test Part B (TMT-B; Lezak et al., 2012) to assess the executive function “shifting” (Miyake et al., 2000) and the Executive Control Task (Baller et al., 2006) to assess working memory or the executive function “updating” (Miyake et al., 2000). Furthermore, three different parallel versions of the Paired-Associates Learning task (Baller et al., 2006) were used to assess visual long-term memory, while the German version (Härting et al., 2000) of the Logical Memory subtest (Story Recall, story A, no delayed recall) from the Wechsler Memory Scale-Revised (WMS-R; Wechsler, 1987) was applied to assess verbal long-term memory. The Trail Making Test Part A (TMT-A; Lezak et al., 2012) and the Digit Symbol Substitution Task (DSST) from Wechsler Adult Intelligence Scale-Revised (WAIS-R; Wechsler, 1981) were used to assess information processing speed. Finally, the Age Concentration Test A with three different parallel versions (Gatterer, 2008) was applied to assess attention (the original test was adapted such that the test result was calculated as “number of correct figures” divided by “time”). Results of training-induced adaptations in cognitive parameters were presented in Eggenberger et al. (2015a).

Four *physical performance parameters* were selected to identify associations with HRV indices. These comprised the 6-MWT walking distance, the total score of the Short Physical Performance Battery (SPPB), the five chair-rise test time (5-CR), and the balance test score. The 6-MWT was used to assess functional aerobic endurance performance (ATS Committee on Proficiency Standards for Clinical Pulmonary Function Laboratories, 2002). Participants had to walk as far as possible on a 30-m walking course within 6 min. Lower-extremity functioning and general functional fitness were measured with the SPPB, which includes a balance test, a 3-m-walk test, and the 5-CR (Guralnik et al., 1994). Thereby, the 3-m-walk test assesses preferred walking speed, whereas the 5-CR reflects functional leg strength. The standard SPPB balance test was extended by adding two increased levels of difficulty to avoid potential ceiling effects (Eggenberger et al., 2015b).

Six temporal and spatial *gait parameters* were selected to identify associations with HRV indices, including “step time variability” and “step length variability” at preferred walking speed, as well as gait velocity under single- (ST) and dual-task (DT) conditions and at preferred and fast walking speeds: “velocity preferred-ST,” “velocity fast-ST,” “velocity preferred-DT,” and “velocity fast-DT.” The parameters were chosen based on findings, which have demonstrated their relation to brain functional or structural aspects (Tian et al., 2017; Ghanavati et al., 2018; Lucas et al., 2018). The GAITRite electronic walkway (CIR Systems, Havertown, PA, USA) with Platinum Version 4.0 software was used to assess gait parameters. A detailed description of the applied gait assessment procedure and the results of training-induced adaptations in gait and physical performance parameters have been reported in Eggenberger et al. (2015b).

### HRV Data Processing

Electrocardiogram (ECG) and R-peaks were recorded at a sampling rate of 1,000 Hz, providing a 1-ms temporal resolution for each R–R interval (Williams et al., 2017). Polar ProTrainer 5 software (Polar Electro Oy, Kempele, Finland) was used to transmit the recorded data from the watch to the computer. The first 30 s of the recording were deleted to reduce initial motion artifacts, and the subsequent 5 min were used for the analyses. Two different methods were applied for artifact reduction: (a) interpolation of artifacts with Kubios HRV analysis software (version 2.2, Kubios Oy, Kuopio, Finland; settings: custom = 0.2, interpolation rate = 4 Hz); and (b) deletion of artifacts with Matlab software (version R2016b, MathWorks Inc., Natick, MA, USA), i.e., R–R intervals <200 ms, >2,000 ms, or >20% interval to interval deviation were removed. Based on the criteria above, the artifact examination with Matlab software yielded a mean of 1.2% (±3.1%) artifacts per R–R interval recording (i.e., 5-min measurements). R–R interval time series with ≥80% non-artifact R–R intervals were used for further analyses (Peltola, 2012), while the time series below this value (and the associated HRV indices) were replaced with the group mean value. This procedure resulted in two 5-min measurements that needed to be replaced (1.1% of all measurements).

After the artifact correction in Kubios or deletion in Matlab, respectively, the HRV processing was performed according to the procedure described by Gąsior et al. (2016). Thereby, data was imported into Kubios where the HRV indices were extracted. Detrending (settings: method = smoothn priors, Lambda = 1,000, f_c_ = 0.029 Hz) and power spectral analysis by means of fast Fourier transformation (settings: HF range = 0.15–0.4 Hz, window width = 60 s, window overlap = 50%) were applied. In order to remove bias on HRV resulting from varying resting heart rates, the time and frequency-domain indices were normalized with respect to the mean R–R interval using the following formulae: SDNN/mean R–R^2^, pNN50/mean R–R^7^, RMSSD/mean R–R^3^, LF power/mean R–R^2^, HF power/mean R–R^4^, and LF to HF power ratio/mean R–R^2^ (Sacha, 2013; Gąsior et al., 2016). SD1 and SD2 were not normalized, as no corresponding procedure was described by the authors.

### Statistical Analyses

We performed one-way analyses of variance (ANOVA) to determine group differences at baseline (pre-training). Multiple linear regression analyses (stepwise backward method) were applied to analyze if the HRV indices (as individual dependent variables) are associated with specific cognitive, physical, or gait parameters at baseline (pre-training). Initially, the cognitive, physical, and gait parameters were analyzed separately with one backward regression analysis for each of these three groups of predictors. Based on these initial calculations, three HRV indices in which most of the variance could be explained were selected and used for all further analyses. Furthermore, the five cognitive, physical, and gait parameters that explained most of the variance in HRV were selected and combined in one final regression analysis to predict the HRV indices. Thereby, age and body mass index (BMI) of the participants were included as additional predictors in the regression model. Hence, for each of the three selected HRV indices, four multiple linear regression analyses were calculated in total. Due to the use of preplanned hypotheses and the exploratory nature of this study, we did not adjust *p*-values to account for possible multiple comparison effects (Perneger, 1998; Streiner and Norman, 2011; Armstrong, 2014). This procedure was applied: (a) to the data set edited with artifact interpolation in Kubios; and (b) to the data set edited by deletion of artifacts in Matlab to evaluate if the different data processing methods would produce comparable results in terms of which predictors are most important.

The effects of the 6-month training intervention on the three selected HRV indices were analyzed by means of multiple linear regression analysis with planned comparisons, including orthogonal contrasts and polynomial trend coding. Based on our predefined hypotheses, we produced contrast-coding variables. In the first contrast, we compared the two combined cognitive–motor training intervention groups (DANCE and MEMORY) with the exclusively physical training group (PHYS). In the second contrast, the two cognitive–motor training intervention groups were compared against each other (DANCE vs. MEMORY). Effect code variables were created for every group’s individuals to account for subject effects. Missing HRV data related to measurement artifacts (see “HRV Data Processing” section) and from participants who attended the full testing period but missed particular test items due to health constraints or personal obligations were replaced by the mean value of the group at the respective time point of measurement and are specified in the results section (simple mean imputation method). Supplementary mediation analyses were performed (Field, 2018; Bherer et al., 2019) to evaluate if changes in cognitive executive functions (TMT-B and Executive Control Task) mediated exergame training (DANCE) effects on changes in HRV indices. All statistical analyses were executed with IBM SPSS Statistics software for Windows (version 25, IBM Corp., Armonk, NY, USA) with a significance level of *α* = 0.05. For mediation analyses, the PROCESS macro for SPSS was used (Hayes, 2013). Effect sizes in multiple linear regression analyses were defined as small for *R^2^* = 0.02 and Δ*R*^2^ = 0.01, medium for *R^2^* = 0.13 and Δ*R*^2^ = 0.06, and large for *R^2^* ≥ 0.26 and Δ*R*^2^ ≥ 0.14. Magnitude of effect size *r* (derived from ANOVA) was considered as small for *r* = 0.10, medium for *r* = 0.30, and large for *r* ≥ 0.50 (Cohen, 1988).

## Results

Seventy out of 89 participants completed the full 6-month intervention (21.4% attrition) and were included for statistical analyses. For a detailed description of the participant flow, including time points and reasons for dropouts, see Eggenberger et al. (2015b). The number of dropouts per intervention group was equally distributed, and therefore, the final analyses were conducted using only the data of participants who completed the entire 6-month intervention. Missing HRV data from the included 70 participants comprised eight values at pretest (2 in DANCE, 4 in MEMORY, 2 in PHYS), 2 values at the 3-month test (1 in MEMORY, 1 in PHYS), and 11 values at the 6-month test (5 in DANCE, 5 in MEMORY, 1 in PHYS). No participant had more than one missing value, except for one participant in the MEMORY group showing two missing values (pre- and 6-month tests). Baseline demographic characteristics of all individuals of the three intervention groups are illustrated in Table 1.

**Table 1:** Baseline demographic characteristics.

Variable	Dance	Memory	Phys	*p*, two-tailed
*n*	24	22	24
Sex, female	14, 58.3%	16, 72.7%	15, 62.5%	0.592
Age, years	77.3 (6.3)	78.5 (5.1)	80.7 (4.8)	0.095
Height, cm	165.1 (7.7)	163.9 (8.5)	162.0 (8.4)	0.458
Weight, kg	75.8 (12.3)	73.6 (9.4)	68.7 (13.7)	0.116
BMI, kg/m^2^	23.0 (3.6)	22.4 (2.2)	22.2 (3.3)	0.138
MMSE	28.4 (1.4)	28.3 (1.2)	28.0 (1.7)	0.543
Hypertension	14, 58.3%	14, 63.6%	13, 54.2%	0.815
Diabetes mellitus	4, 16.7%	3, 13.6%	2, 8.3%	0.693

*Notes: Data are numbers or means (standard deviation in brackets). Numbers for hypertension and diabetes mellitus are based on self-reported data from health questionnaire. *P*-values are based on one-way ANOVA. Abbreviations: DANCE, virtual reality video game dancing; MEMORY, treadmill walking with simultaneous verbal memory training; PHYS, treadmill walking; MMSE, Mini Mental State Examination*.

### Cross-sectional Analyses

The three initial regression analyses that were performed separately for each group of predictors (i.e., cognitive, physical, or gait parameters, respectively) revealed that most variance could be explained in the three HRV indices SDNN, RMSSD, and HF power. Therefore, these three indices were included for further analyses and will be reported. They also represent the most commonly used HRV indices (Ernst, 2017). Representative results of the three separate initial backward regression analyses are presented for the RMSSD index in Table 2. Similar results were found for SDNN and HF power and are therefore not reported. Based on these results, for analysis: (a), including the data set edited with artifact interpolation in Kubios, the final regression analysis was performed with the following seven parameters: TMT-B, Story Recall, step length variability (at preferred single task walking speed), SPPB, 5-CR, age, and BMI. The corresponding results are illustrated in Table 3. For analysis: (b), comprising the data set edited by deletion of artifacts in Matlab, the final regression analysis included TMT-B, Story Recall, gait velocity (at preferred dual task walking speed), SPPB, 5-CR, age, and BMI. From these analyses, the models that included only significant parameters (or trends to significance, i.e., *p* < 0.10, if no significance was attained) were selected. The results from analysis (a) (Kubios) vs. (b) (Matlab) revealed no differences in which parameters were significantly contributing to the models. Therefore, only the results based on the data set with artifact interpolation in Kubios are reported and this data set will be used for the following longitudinal analyses.

**Table 2:** Initial linear regression models to predict HRV with three separate groups of predictors, including cognitive, physical, and gait parameters, respectively.

Predictors (RMSSD = dependent variable)		*b (95% CI)*	*SE B*	*β*	*t*	*p*, two-tailed (predictor)	*R^2^* (model)	*p*, two-tailed (model)
Cognitive parameters	TMT-B	−2.69 × 10^−10^ (–4.71 × 10^−10^,–6.80 × 10^−11^)	1.01 × 10^−10^	−0.331	−2.670	0.010*	0.107	0.022*
	Story Recall	−3.58 × 10^−9^ (–7.41 × 10^−9^,–2.44 × 10^−10^)	1.92 × 10^−9^	−0.232	−1.869	0.066^t^		
	Constant	1.23 × 10^−7^ (6.94 × 10^−8^, 1.76 × 10^−7^)	2.67 × 10^−8^		4.600	<0.001*		
Physical parameters	5-CR	4.44 × 10^−9^ (6.04 × 10^−10^, 8.28 × 10^−9^)	1.92 × 10^−9^	0.393	2.310	0.024*	0.077	0.069^t^
	SPPB	6.24 × 10^−9^ (–3.04 × 10^−9^, 1.55 × 10^−8^)	4.65 × 10^−9^	0.228	1.342	0.184		
	Constant	−6.28 × 10^−8^ (–1.97 × 10^−7^, 7.16 × 10^−8^)	6.73 × 10^−8^		−0.933	0.354		
Gait parameters	Step length variability (pref. ST)	−5.51 × 10^−9^ (–1.78 × 10^−8^, 6.73 × 10^−9^)	6.14 × 10^−9^	−0.120	−0.899	0.372	0.015	0.600
	Velocity (pref. ST)	−2.15 × 10^−10^ (–7.52 × 10^−10^, 3.22 × 10^−10^)	2.69 × 10^−10^	−0.107	−0.800	0.427		
	Constant	9.71 × 10^−8^ (1.05 × 10^−8^, 1.84 × 10^−7^)	4.34 × 10^−8^		2.238	0.029*		

Notes: Representative results for RMSSD as dependent variable are presented. Similar results were found for SDNN and HF power (not reported). HRV based on data set with artifact interpolation in Kubios. The following predictors were included to run the three backward regression analyses: cognitive parameters: TMT-B, Story Recall, DSST, Age Concentration Test A, Executive Control Task, Paired-Associates Learning task, TMT-A; physical parameters: 5-CR, SPPB, Balance, 6-MWT; gait parameters: step length variability pref. ST, velocity pref. ST, velocity fast ST, velocity pref. DT, step time variability pref. ST. Therefrom, the models that included the last two parameters retained in the backward regression analyses are presented here. Out of these six parameters, the five most significant parameters were selected and combined together with age and BMI in the final linear regression model (Table 3). Abbreviations: BMI, body mass index; DSST, Digit Symbol Substitution Task; DT, dual task; HF power, absolute power of high-frequency band (0.15–0.40 Hz); pref., preferred walking speed; RMSSD, root mean square of successive R–R interval differences; SDNN, standard deviation of N–N intervals; SPPB, Short Physical Performance Battery; ST, single task; TMT-A/B, Trail Making Test Part A/B; 5-CR, five chair rises; 6-MWT, 6-min-walk test; *p < 0.05, significant; ^t^*p* < 0.10, trend.

**Table 3:** Final linear regression models to predict HRV indices with combined cognitive, physical, and gait parameters.

HRV indices (dependent variables)	Predictors	*b (95% CI)*	*SE B*	*β*	*t*	*p*, two-tailed (predictor)	*R^2^* (model)	*p*, two-tailed (model)
SDNN	TMT-B	−1.37 × 10^−7^ (–2.47 × 10^−7^,–2.64 × 10^−8^)	5.53 × 10^−8^	−0.287	−2.472	0.016*	0.082	0.016*
	Constant	8.39 × 10^−5^ (6.93 × 10^−5^, 9.85 × 10^−5^)	7.33 × 10^−6^		11.442	<0.001*		
RMSSD	TMT-B	−2.14 × 10^−10^ (–4.01 × 10^−10^,–2.79 × 10^−11^)	9.34 × 10^−11^	−0.263	−2.294	0.025*	0.121	0.013*
	5-CR	2.78 × 10^−9^ (1.88 × 10^−10^, 5.37 × 10^−9^)	1.30 × 10^−9^	0.246	2.141	0.036*		
	Constant	4.77 × 10^−8^ (9.86 × 10^−9^, 8.56 × 10^−8^)	1.90 × 10^−8^		2.515	0.014*		
HF power	TMT-B	−6.81 × 10^−12^ (–1.34 × 10^−11^,–2.55 × 10^−13^)	3.28 × 10^−12^	−0.238	−2.074	0.042*	0.119	0.015*
	5-CR	1.05 × 10^−10^ (1.43 × 10^−11^, 1.96 × 10^−10^)	4.56 × 10^−11^	0.266	2.310	0.024*		
	Constant	3.78 × 10^−10^ (–9.53 × 10^−10^, 1.71 × 10^−9^)	6.67 × 10^−10^		0.566	0.573		

Notes: HRV based on data set with artifact interpolation in Kubios. The following predictors were included to run the backward regression analysis: TMT-B, Story Recall, step length variability (at preferred single task walking speed), SPPB, 5-CR, age, and BMI. Therefrom, the models that included only significant parameters (or trends to significance, i.e., *p* < 0.10, if no significance was attained) were selected and are presented here. Abbreviations: BMI, body mass index; HF power, absolute power of high-frequency band (0.15–0.40 Hz); RMSSD, root mean square of successive R–R interval differences; SDNN, standard deviation of N–N intervals; SPPB, Short Physical Performance Battery; TMT-B, Trail Making Test Part B; 5-CR, five chair rises; *p < 0.05, significant; ^t^*p* < 0.10, trend.

### Longitudinal Analyses

At baseline (pre-training), we found no significant differences of any of the HRV indices between the three intervention groups (SDNN: *F*_(2,69)_ = 0.039, *p* = 0.962, two-tailed, *r* = 0.03; RMSSD: *F*_(2,69)_ = 0.824, *p* = 0.443, two-tailed, *r* = 0.15; HF power: *F*_(2,69)_ = 0.242, *p* = 0.786, two-tailed, *r* = 0.08). The *linear global time effect*, which represents the effect when the entire sample across all three intervention groups is merged, indicated a significant increase in SDNN from pre-training to 6-month test (*F*_(1,134)_ = 4.81, *p* = 0.015 one-tailed, Δ*R*^2^ = 0.020). For the other two HRV indices (RMSSD and HF power), no significant linear global time effect was found.

#### Differences Between Cognitive–Motor and Exclusively Physical Training (First Contrast)

The *first contrast*, i.e., the cognitive–motor intervention groups (DANCE and MEMORY) vs. the exclusively physical intervention group (PHYS), yielded a significant linear time×intervention interaction for SDNN (*F*_(1,134)_ = 5.00*, p* = 0.014 one-tailed, Δ*R*^2^ = 0.020) and a trend to significance for RMSSD (*F*_(1,134)_ = 2.68, *p* = 0.052 one-tailed, Δ*R*^2^ = 0.007). These interactions reflect an increase in the two HRV indices in the cognitive–motor groups, while PHYS remained unchanged.

#### Differences Between the Two Cognitive–Motor Training Groups (Second Contrast)

**Figure 2:**
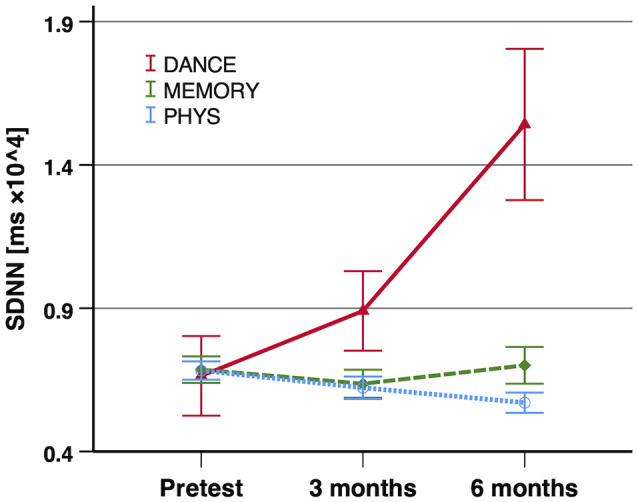
Exercise training-induced adaptations in SDNN. Notes: significant improvements of SDNN were shown in the first contrast (DANCE/MEMORY vs. PHYS; *p* = 0.014 one-tailed, Δ*R*^2^ = 0.020) and in the second contrast (DANCE vs. MEMORY; *p* = 0.002 one-tailed, Δ*R*^2^ = 0.035). Error bars indicate ± standard error of the mean. Abbreviations: SDNN, standard deviation of N–N intervals; DANCE, virtual reality video game dancing; MEMORY, treadmill walking with simultaneous verbal memory training; PHYS, treadmill walking.

**Figure 3:**
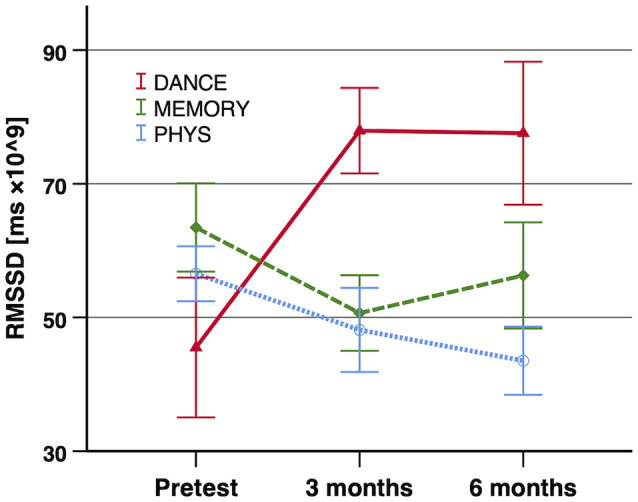
Exercise training-induced adaptations in RMSSD. Notes: significant improvements of RMSSD were shown in the first contrast (DANCE/MEMORY vs. PHYS; trend *p* = 0.052 one-tailed, Δ*R*^2^ = 0.007) and in the second contrast (DANCE vs. MEMORY; *p* = 0.017 one-tailed, Δ*R*^2^ = 0.012). Error bars indicate ± standard error of the mean. Abbreviations: RMSSD, root mean square of successive R–R interval differences; DANCE, virtual reality video game dancing; MEMORY, treadmill walking with simultaneous verbal memory training; PHYS, treadmill walking.

**Figure 4:**
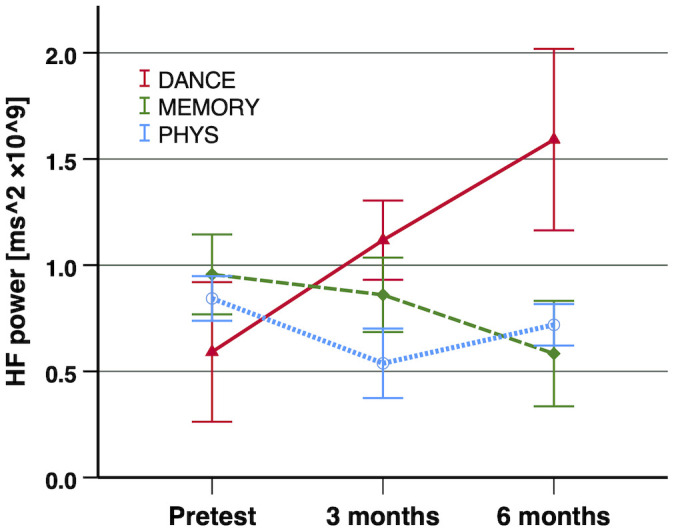
Exercise training-induced adaptations in HF power. Notes: a significant improvement of HF power was shown in the second contrast (DANCE vs. MEMORY; *p* = 0.011 one-tailed, Δ*R*^2^ = 0.013). Error bars indicate ± standard error of the mean. Abbreviations: HF power, absolute power of high-frequency band (0.15–0.4 Hz); DANCE, virtual reality video game dancing; MEMORY, treadmill walking with simultaneous verbal memory training; PHYS, treadmill walking.

In the *second contrast*, the two cognitive–motor intervention groups (DANCE vs. MEMORY) were compared. For all three investigated HRV indices, a significant linear time × intervention interaction was evident: SDNN (*F*_(1,134)_ = 8.69, *p* = 0.002 one-tailed, Δ*R*^2^ = 0.035), RMSSD (*F*_(1,134)_ = 4.63, *p* = 0.017 one-tailed, Δ*R*^2^ = 0.012), and HF power (*F*_(1,134)_ = 5.47, *p* = 0.011 one-tailed, Δ*R*^2^ = 0.013). These interactions reflect an increase in all three HRV indices in DANCE, while MEMORY remained unchanged. The longitudinal development of the three HRV indices, from baseline (pre-training) to 6-month test, is illustrated in Figures 2–4, respectively.

#### Supplementary Analysis: Mediation of Exergame Training Effects on Changes in HRV Through Changes in Cognitive Executive Functions

No significant indirect effect of training intervention (i.e., DANCE vs. MEMORY/PHYS) on changes in standardized HRV indices (z-scores) through mediation of changes in standardized cognitive executive functions was evident: change in SDNN mediated by change in TMT-B and Executive Control Task, respectively: *b* = 0.034, 95% BCa CI [–0.078, 0.193] and *b* = 0.019, 95% BCa CI [–0.041, 0.094]; change in RMSSD mediated by change in TMT-B and Executive Control Task, respectively: *b* = –0.015, 95% BCa CI [–0.119, 0.060] and *b* = –0.040, 95% BCa CI [–0.159, 0.056]; and change in HF power mediated by change in TMT-B and Executive Control Task, respectively: *b* = –0.004, 95% BCa CI [–0.071, 0.059] and *b* = –0.031, 95% BCa CI [–0.144, 0.046]. Significant mediation was indicated when confidence intervals did not include zero (Field, 2018).

## Discussion

Our study *aimed*: (1) to investigate which cognitive, physical, and gait parameters explain the variance in HRV indices; and (2) to investigate whether cognitive–motor training induces different adaptations in HRV indices compared to exclusively physical training. The two *main findings* were that 8–12% of variance in each of the selected HRV indices (SDNN, RMSSD, and HF power) was explained by cognitive executive functions (TMT-B) and leg strength (5-CR, inversely related). Furthermore, our results indicate that 6 months of exergame training (DANCE) markedly increased all three HRV indices, while DT treadmill walking (MEMORY) and exclusively physical training (PHYS) did not affect HRV in older adults.

### Cross-sectional Analyses

To the best of our knowledge, this is the first study to investigate a combination of different parameters of cognitive, physical, and gait performance as predictors of HRV in older adults. We hypothesized that variance in HRV is explained by: (a) cognitive parameters related to executive functions and verbal memory; (b) physical parameters related to physical functioning, aerobic endurance, and leg strength; and (c) gait parameters related to executive functions.

Cognitive *executive functioning* (TMT-B) was the most prominent significant predictor of variance in all three assessed HRV indices (SDNN, RMSSD, and HF power). This finding is in line with previous cross-sectional studies and a recent meta-analysis, which demonstrated that HRV indices may mirror executive functions in several age groups (*N* = 14,347) and that this association becomes even stronger in older people (Holzman and Bridgett, 2017). Similarly, higher performance in dual tasks with great demands on executive functioning was closely related to increased parasympathetic activity in very fit older adults (i.e., Master Athletes; Dupuy et al., 2018). These and our own findings highlight the role of fitness level on cardiac autonomic control and executive functioning. Additionally, the results support the “neuro-visceral integration model,” which proposes that cognitive executive functions are linked to cardiac autonomic regulation and both are controlled by the prefrontal cortex together with other brain areas (Thayer et al., 2009, 2012).

*Verbal long-term memory* (story recall) did not significantly contribute to variance in HRV in our study. In contrast, verbal learning and memory were linked with very-low-frequency (VLF) HRV from 24-h measurements in 416 middle-aged male twins (mean age 55 ± 2.9 years; Shah et al., 2011). Equally, Frewen et al. (2013) reported that mainly the memory recall and language sub-domains of the Montreal Cognitive Assessment (MoCA) were responsible for the relationship of the MoCA with HRV (i.e., SDNN, LF, and LF/HF ratio) in a large cohort of older adults (*N* = 4,763, mean age 61.7 ± 8.3 years). Interestingly, patients with mild cognitive impairments (MCI) demonstrate parasympathetic deficits, i.e., reduced HF power, and patients with greater autonomic dysfunction exhibit more pronounced neuropsychological deficits, probably due to early structural and functional neural alterations of the central autonomic network related to Alzheimer’s disease (Collins et al., 2012).

*Leg strength* (5-CR), but not overall *functional fitness* (SPPB total score, comprising gait speed, 5-CR, and balance), emerged as a significant inversely related predictor of variance in the two parasympathetic HRV indices RMSSD and HF power. Hence, better leg strength (i.e., shorter time on the 5-CR test) was associated with lower or worse HRV indices and *vice versa*. Comparably, Matos et al. (2020) recently reported that SDNN and RMSSD were inversely correlated with hand-grip strength in 28 older adults (*r* = –0.30 and –0.23, respectively); although the correlation was not statistically significant, the result seems worth mentioning due to the medium effect size. Hand-grip strength dynamometry and functional leg strength assessments are both valid tools to measure muscle strength and are feasible for sarcopenia screening in home settings of community-dwelling older adults (Mijnarends et al., 2013). Sarcopenia is a condition dependent on the quality of the mechanism of corticospinal interaction and central drive to skeletal muscle (Gennaro et al., 2020) and is characterized by low muscle strength, muscle mass, and physical performance and accompanied by increased cardiac risk (Cruz-Jentoft et al., 2019). To our knowledge, no study has previously investigated the specific association of leg strength with HRV. Therefore, further research is warranted to substantiate or refute this finding. Nevertheless, our result which showed that overall functional fitness did not explain variance in HRV contradicts previous studies with older adults. For instance, Graham et al. (2019) recorded long-term HRV (approximately 2.5 h) during ADL with a wrist-worn photoplethysmography (PPG) sensor. They observed significant correlations of HRV with two tests of physical functioning, including the SPPB and the Timed Up and Go test (TUG) in 52 older adults between 65 and 95 years of age. In a 5-year longitudinal assessment among 985 US older adults (mean age 71 ± 5 years), higher total leisure-time activity, as well as walking distance and pace, was prospectively related to more favorable HRV indices SDNN and ultra-low-frequency power (ULF power, <0.003 Hz) recorded over 24 h (Soares-Miranda et al., 2014). Comparably, older adults (mean age 75.7 ± 0.2 years) who followed a long-term sportive lifestyle exhibited increased 5-min global HRV (SDNN) and parasympathetic HRV indices (e.g., RMSSD and HF power) compared to their active but non-sportive counterparts; Buchheit et al., 2004). Moreover, older adults affected by sarcopenia showed reduced modulation of the parasympathetic autonomic nervous system as assessed with 5-min time-domain and nonlinear HRV indices (Freitas et al., 2018). Similarly, a shift toward sympathetic predominance was evident in frail elderly women, suggesting that HRV monitoring may be of value for the prevention, diagnosis, and treatment of the frailty syndrome (Katayama et al., 2015).

*Gait performance*, particularly step length variability at preferred walking speed, was another predictor that did not make a significant contribution to the combined regression models in our study. This outcome conforms with the only previous investigation of associations of HRV with measures of gait from Aerts et al. (2009). The authors did not find any significant correlation of gait performance and HRV in their sample of healthy older adults and Parkinson’s disease patients (*n* = 15 and 18, respectively). Nevertheless, we suggest that future studies may still further investigate this interrelation, since gait performance and gait control are interrelated not only with cognitive executive functions but also with prefrontal and cingulate cortex activity (Cohen et al., 2016; Sakurai et al., 2017). Therefore, one might have expected to find an association of gait parameters with HRV indices, based on their shared reliance on partially the same brain areas, including the prefrontal and cingulate cortices (Thayer et al., 2009; Tian et al., 2017; Ghanavati et al., 2018; Lucas et al., 2018).

In contrast to previous research, *aerobic fitness* (6-MWT) was not a relevant predictor for HRV in our regression models. Dorey et al. (2019) reported higher HF power in aerobically fit compared to aerobically unfit older adults. Thereby, aerobic fitness was determined as VO_2_ peak in a graded cycle ergometer test. Also in senior athletes, parasympathetic HRV was significantly higher than in sedentary older adults and correlated positively with maximal aerobic capacity (Yataco et al., 1997). Because resting heart rate was also lower in older adults with better physical fitness (Buchheit et al., 2004; Dupuy et al., 2018), our findings may be linked to the fact that we have normalized HRV indices for average R–R interval duration to adjust for interindividual differences in resting heart rate. This procedure appears to be crucial to improving reproducibility and comparability of HRV recordings, since already a minimal change in heart rate by 1 bpm results in considerable alterations of HRV indices by 16.5% on average (Gąsior et al., 2016). Comparably, Monfredi et al. (2014) suggested that changes in HRV due to altered morbidity and mortality are substantially associated with concurrent changes in heart rate. Therefore, the authors emphasized that the results of studies failing to properly adjust HRV for interindividual heart rate differences would have to be reevaluated. Nonetheless, also in a supplementary analysis, using the non-normalized HRV data of our study (results not reported), the relevance of the 6-MWT as a predictor of HRV did not increase.

### Longitudinal Analyses

We further hypothesized that exercise training-induced adaptations in HRV indices primarily occur in cognitive–motor training interventions (i.e., DANCE and MEMORY) and particularly in DANCE, which includes training aspects of executive functioning. We indeed found specific improvements of HRV indices after 6 months of exergame training (DANCE), but not after DT treadmill walking (MEMORY) and following exclusively physical training (PHYS). Since baseline values in nine different tests of cognitive performance were equal among all three intervention groups, as reported previously (Eggenberger et al., 2015a), we exclude the possibility that unequally distributed factors related to cognitive and brain health (e.g., cerebrovascular disease, obesity, hypertension, and diabetes mellitus) may have affected this outcome. The small to medium effect size of our exergame intervention on HRV is in the same range of effects commonly found in aerobic endurance training interventions in healthy older adults, as reported in a meta-analysis (Raffin et al., 2019). Therefore, the effects in our study appear to be domain-specific and of clinical and practical importance (Durlak, 2009). To our knowledge, the effect of exergame training on HRV indices has not been investigated previously in healthy older adults. Nevertheless, our results are coherent with two recent trials applying exergame paradigms in individuals with chronic stroke (Sampaio et al., 2016) and women with fibromyalgia (Villafaina et al., 2020). Sampaio et al. (2016) performed 6 weeks (20 sessions) of a virtual reality-based dance video game in a small sample of eleven patients with chronic hemiparetic stroke (age 61.7 ± 4.3 years) and found improvements in autonomic modulation assessed with LF power, HF power, and LF/HF HRV indices which were accompanied by increased VO_2_max (computed from the YMCA submaximal cycle ergometer test). Meanwhile, Villafaina et al. (2020) recently conducted a randomized controlled trial including 50 women with fibromyalgia. The intervention group (age 54.0 ± 8.5) completed two weekly 1-h exergame sessions over 24 weeks and displayed improved SDNN and other HRV indices compared to the passive control group.

Supplementary mediation analysis revealed that changes in cognitive executive functions did not mediate exergame training effects (DANCE vs. MEMORY/PHYS) on changes in HRV. Nevertheless, such a mediation effect appeared possible since the exergame training in this study particularly improved cognitive executive functions as reported previously (Eggenberger et al., 2015a) and similar findings were summarized in recent meta-analytic and review articles (Stanmore et al., 2017; Stojan and Voelcker-Rehage, 2019). Moreover, another study demonstrated that behavioral training effects on cognitive executive functions are reflected in brain functional modulations in the prefrontal cortex area as assessed with functional near-infrared spectroscopy (fNIRS) during challenging walking (Eggenberger et al., 2016). Thereby, the prefrontal cortex mediates the association of cognitive executive functions with HRV (Yoo et al., 2018). Neuroimaging and pharmacological studies have demonstrated a link between HRV and prefrontal neural function (Thayer et al., 2009, 2012). Therefore, the potential role of changes in cognitive executive functions as a mediator of specific exergame training effects on HRV warrants further investigation in order to substantiate or refute this finding. In summary, together with the cross-sectional associations of HRV with cognitive executive functions found in the present study, the outcome of our longitudinal analyses does further support the notion that HRV particularly mirrors executive functioning.

The two intervention groups MEMORY and PHYS both comprised treadmill walking as an aerobic exercise-training component. However, they did not increase HRV indices, which conflicts with previous research. Recent meta-analytic results show that studies including exclusively aerobic-type training mostly demonstrated improvements in HRV in older adults and particularly in the global HRV index SDNN (Raffin et al., 2019). In some trials, aerobic training also specifically increased the parasympathetic mediated HRV indices (i.e., RMSSD and HF power) with a concomitant enhancement of cognitive executive functions (Albinet et al., 2010, 2016). Similarly, in a study including 118 sedentary, overweight, hypertensive women (mean age 64.8 ± 4.2 years), 6 months of low-to-moderate intensity aerobic training raised parasympathetic HRV indices, despite VO_2_max remaining unchanged (Earnest et al., 2012). Raffin et al. (2019) proposed in their meta-analysis that long-term aerobic exercise interventions with a high training frequency, i.e., four or more sessions per week over several months, are the key to positively affecting HRV in older adults, rather than the duration and intensity of the single sessions. It appears that exclusively aerobic exercise-based interventions require a much higher training frequency to induce positive adaptations of HRV, compared to our twice-weekly exergame intervention (DANCE). Therefore, our conflicting result may be the consequence of the relatively low training frequency of aerobic-type exercise in MEMORY and PHYS, i.e., twice weekly, and the relatively low fraction of specific aerobic endurance training volume, i.e., 20 min per session. However, the training still improved aerobic endurance assessed with the 6-MWT as reported elsewhere (Eggenberger et al., 2015b). Furthermore, the opposing result may again be related to normalizing HRV indices with average R–R intervals in our study. Thereby, the normalizing procedure may have leveled out any effects on HRV that could be linked to the possibly lowered resting heart rate after the training intervention. Similar to our exclusively physical training group which comprised strength, balance, and treadmill walking (PHYS), the few available strength training studies appeared not to have an impact on HRV in older adults (Forte et al., 2003; Madden et al., 2006; Gerhart et al., 2017) or even reduced HRV (Melo et al., 2008). The authors suggested that diverging mechanisms mediate beneficial effects of strength compared to aerobic training on the cardiovascular system.

An intriguing finding in the context of our study is the observation from Vesterinen et al. (2016) that HRV has potential to be used as a tool that may be able to help in designing individualized training programs. For instance, it seems that individuals (younger adults) with low HRV values rather respond with increases in endurance performance when high-volume low-intensity training is endorsed. On the opposite, trainees with high initial HRV values respond better to high-intensity training with progressively increasing training volumes and intensities (Vesterinen et al., 2016). In the present study, we developed and applied our training programs in the traditional way, i.e., based on scientific literature, guidelines, and recommendations, in combination with the practical experience of our trainers. Taking this approach means that our programs were prescribed without information on how the older adults have responded to past training. However, from previous research we know that such an approach may lead to success on a group level but might, at the same time, hide interindividual differences in training response because not all individuals respond in a similar way. Large individual differences in response to the same training program are not uncommon in research literature (Bouchard and Rankinen, 2001; Hautala et al., 2003; Vollaard et al., 2009), which points out the necessity of designing individualized training programs. Therefore, further research is warranted to investigate the potential of baseline HRV assessments as a means to individualize training prescription in older adults.

### Strengths and Limitations

A methodological strength of the presented study was the comparably large number of participants and the long training period of 6 months. The following limitations need to be considered. First, the conclusions from this study are limited to the sample population that was involved, i.e., physically and cognitively healthy older adults. It may be argued that training effects could have been even higher in physically and cognitively impaired older persons. This assumption is based on the exercise-training principle “initial values,” which states that exercise training-induced improvements will be largest in persons with lower initial fitness levels (Ammann et al., 2014). Thereby, it might be particularly interesting to assess effects of exergame training on HRV in healthy older adults compared to patients with cerebrovascular pathology which is associated with reduced executive functioning (Poulin et al., 2012). Second, although participants were blinded in relation to the hypothesized study outcome, it was not possible to blind the investigators as they were involved in supervising and conducting training and test sessions. Finally, the chosen simple mean imputation method provides valid estimations of intervention effects but lowers variability and *p*-values (Dziura et al., 2013). Variability in the data including imputed missing values was on average 5.5% (±4.4%) lower compared to the data with missing values. Considering the overall large variability in the HRV data (standard deviation in % of mean HRV values: with imputed values = 116.2% ± 70.3%, with missing values = 123.1% ± 74.3%) leads us to conclude that the imputed missing values had a negligible effect on the presented results.

## Conclusion

The present study provides important novel information supporting the predictive value of HRV indices as markers of cognitive health in older adults. To our knowledge, this is the first investigation of a combination of different parameters of cognitive, physical, and gait performance as predictors of HRV indices (SDNN, RMSSD, and HF power) representing global and parasympathetic aspects of the autonomic nervous system. Thereby, we found in the cross-sectional analysis that 8–12% of variance in each of the three HRV indices was significantly explained by cognitive executive functions and, inversely related, by leg strength. Furthermore, in the longitudinal analysis we identified that 6 months of exergame training (DANCE) markedly increased all three HRV indices, while DT treadmill walking (MEMORY) and exclusively physical training (PHYS) did not have any effects on HRV. Since the exergame training in this study specifically improved cognitive executive functioning in older adults, as reported previously (Eggenberger et al., 2015a), the outcomes of our cross-sectional and longitudinal analyses are highly coherent. We conclude that mainly cognitive executive functions are associated with HRV indices and, notably, that exergame training represents an effective training strategy to improve global and parasympathetic autonomic nervous system activities in older adults. Therefore, regularly assessing HRV in older citizens could be particularly beneficial to monitor cognitive health and provide indications for preventative exercise-training measures. Future investigations are warranted to strengthen these results and could include other potentially supplementary predictors of HRV, such as measures of brain function and structure. This will further promote our understanding of which factors of cognitive and physical health may be mirrored in HRV measurements in older adults.

## Data Availability Statement

The raw data supporting the conclusions of this article will be made available by the authors, without undue reservation, to any qualified researcher.

## Ethics Statement

The studies involving human participants were reviewed and approved by the ethics committee of the Canton, St. Gallen, Switzerland (EKSG 12/092). The patients/participants provided their written informed consent to participate in this study.

## Author Contributions

PE, TM, and EB contributed with the conception and design of the study. PE recruited the participants. PE conducted and supervised the training and testing sessions. PE, SA, and KK executed the data processing. PE and KK performed the statistical analyses. PE, SA, KK, RR, TM, and EB contributed to data interpretation. PE wrote the first draft of the manuscript. All authors contributed with the manuscript revision and read and approved the submitted version.

## Conflict of Interest

The authors declare that the research was conducted in the absence of any commercial or financial relationships that could be construed as a potential conflict of interest.
